# Establishment of preanalytical conditions for microRNA profile analysis of clinical plasma samples

**DOI:** 10.1371/journal.pone.0278927

**Published:** 2022-12-14

**Authors:** Kuno Suzuki, Tatsuya Yamaguchi, Masakazu Kohda, Masami Tanaka, Hiroyuki Takemura, Mitsuru Wakita, Yoko Tabe, Shunsuke Kato, Motomi Nasu, Takashi Hashimoto, Shinji Mine, Nobuko Serizawa, Ko Tomishima, Akihito Nagahara, Takahisa Matsuda, Taiki Yamaji, Shoichiro Tsugane, Yutaka Saito, Hiroyuki Daiko, Takaki Yoshikawa, Ken Kato, Takuji Okusaka, Takahiro Ochiya, Yusuke Yamamoto, Shoji Yotsui, Takashi Yamamoto, Tomoyuki Yamasaki, Hiroshi Miyata, Masayoshi Yasui, Takeshi Omori, Kazuyoshi Ohkawa, Kenji Ikezawa, Tasuku Nakabori, Naotoshi Sugimoto, Toshihiro Kudo, Keiichi Yoshida, Masayuki Ohue, Takashi Nishizawa

**Affiliations:** 1 Healthcare Business Department, PFDeNA, Inc., Tokyo, Japan; 2 Department of Clinical Laboratory, Juntendo University Hospital, Tokyo, Japan; 3 Department of Clinical Oncology, Juntendo University Graduate School of Medicine, Tokyo, Japan; 4 Department of Esophageal and Gastroenterological Surgery, Juntendo University Graduate School of Medicine, Tokyo, Japan; 5 Department of Gastroenterology, Juntendo University Graduate School of Medicine, Tokyo, Japan; 6 Cancer Screening Center, National Cancer Center Hospital, Tokyo, Japan; 7 Division of Epidemiology, National Cancer Center Institute for Cancer Control, Tokyo, Japan; 8 Division of Cohort Research, National Cancer Center Institute for Cancer Control, Tokyo, Japan; 9 Department of Endoscopy, National Cancer Center Hospital, Tokyo, Japan; 10 Department of Esophageal Surgery, National Cancer Center Hospital, Tokyo, Japan; 11 Department of Gastric Surgery, National Cancer Center Hospital, Tokyo, Japan; 12 Department of Head and Neck, Esophageal Medical Oncology / Department of Gastrointestinal Medical Oncology, National Cancer Center Hospital, Tokyo, Japan; 13 Department of Hepatobiliary and Pancreatic Oncology, National Cancer Center Hospital, Tokyo, Japan; 14 Laboratory of Integrative Oncology, National Cancer Center Research Institute, Tokyo, Japan; 15 Clinical Laboratory, Osaka International Cancer Institute, Osaka, Japan; 16 Department of Gastroenterological Surgery, Osaka International Cancer Institute, Osaka, Japan; 17 Department of Hepatobiliary and Pancreatic Oncology, Osaka International Cancer Institute, Osaka, Japan; 18 Department of Medical Oncology, Osaka International Cancer Institute, Osaka, Japan; 19 Next-generation Precision Medicine Research Center, Osaka International Cancer Institute, Osaka, Japan; Universita degli Studi di Torino, ITALY

## Abstract

The relationship between the expression of microRNAs (miRNAs) in blood and a variety of diseases has been investigated. MiRNA-based liquid biopsy has attracted much attention, and cancer-specific miRNAs have been reported. However, the results of analyses of the expression of these miRNAs vary among studies. The reproduction of results regarding miRNA expression levels could be difficult if there are differences in the data acquisition process. Previous studies have shown that the anticoagulant type used during plasma preparation and sample storage conditions could contribute to differences in measured miRNA levels. Thus, the impact of these preanalytical conditions on comprehensive miRNA expression profiles was examined. First, the miRNA expression profiles of samples obtained from healthy volunteers were analyzed using next-generation sequencing. Based on an analysis of the library concentration, human genome identification rate, ratio of unique sequences and expression profiles, the optimal preanalytical conditions for obtaining highly reproducible miRNA expression profiles were established. The optimal preanalytical conditions were as follows: ethylenediaminetetraacetic acid (EDTA) as the anticoagulant, whole-blood storage at room temperature within 6 hours, and plasma storage at 4°C or -20°C within 30 days. Next, plasma samples were collected from 60 cancer patients (3 facilities × 20 patients/facility), and miRNA expression profiles were analyzed. There were no significant differences in measurements except in the expression of erythrocyte-derived hsa-miR-451a. However, the variation in hsa-miR-451a levels was smaller among facilities than among individuals. This finding suggests that samples obtained from the same facility could show significantly different degrees of hemolysis across individuals. We found that the standardization of anticoagulant use and storage conditions contributed to reducing the variation in sample quality across facilities. The findings from this study could be useful in developing protocols for collecting samples from multiple facilities for cancer screening tests.

## Introduction

MicroRNAs (miRNAs) are small noncoding RNAs comprising 20–25 nucleotides, and miRNAs regulate gene expression by inducing mRNA degradation and inhibiting mRNA transcription into proteins. More than 2,500 miRNAs have been identified previously [1]. It has been reported that the changes in miRNA expression observed in blood are associated with human diseases such as cancer [2–4], cardiovascular disease [5–7], and Alzheimer’s disease [8–10]. Because early detection and treatment of cancer leads to improved survival rates, screening tests are conducted for each type of cancer. However, testing for multiple types of cancer is a physical and financial burden for patients. MiRNA-based liquid biopsy is attracting attention since it is a less invasive method for cancer screening [2–4, 11–16].

Previous studies have reported that the expression levels of some miRNAs can be used to distinguish between individuals with and without cancer [11–14, 16]. Furthermore, the development of technologies for comprehensive data acquisition, such as next-generation sequencing (NGS) and microarrays, has made it possible to obtain the expression profiles of more than 2,500 different miRNAs in a single measurement. Through the identification of cancer-specific markers, cancer and noncancer were able to be distinguished with an area under the receiver operating characteristic (ROC) curve of 0.92–0.98 [2–4, 15]. MiRNA biomarkers are reported to be useful not only in distinguishing the presence or absence of cancer but also in binary classification of certain cancer types, with 95% accuracy for bladder cancer [3] and more than 95% accuracy for six other cancer types. MiRNA biomarkers were also shown to be useful in distinguishing between ovarian cancer and noncancer [4] with 84% accuracy and in distinguishing between ovarian cancer and four nonovarian cancer types with more than 80% accuracy. It has been suggested that a screening test that simultaneously classifies multiple cancer types can be achieved by obtaining a comprehensive miRNA expression profile.

In previous studies on the discovery of cancer-specific markers, miRNAs that showed large fold changes in expression between a certain cancer type and other cancer types were identified as cancer-specific miRNAs [17–20]. Although studies have identified cancer-specific markers, the results regarding the changes in the expression levels of these miRNAs are controversial [21–23]. We speculate that the low reproducibility of the results regarding the changes in the expression levels of the same miRNAs is attributable to differences in the data acquisition methods used among the studies. Since sample collection is typically conducted using different operational methods across each facility, the type of blood collection tubes and the temperature and timing of storage of whole blood and plasma samples are potentially variable between facilities. The inconsistencies in the results regarding cancer-specific marker among studies are thought to be related to these differences in preanalytical conditions. Studies on the type of anticoagulant in blood collection tubes have revealed different expression levels of miRNAs, including hsa-miR-191-5p, hsa-miR-320a, hsa-miR-21-5p and hsa-miR-451a [24]; hsa-miR-21 and hsa-miR-29b [25]; and hsa-miR-16 [26], in samples obtained from ethylenediaminetetraacetic acid (EDTA)-treated plasma and citrate-treated plasma obtained from the same source. Although these studies focused on a limited number of miRNAs, studies on the storage conditions used for whole blood and plasma have reported that some miRNAs show small changes in expression over 24 hours in whole blood [25, 27] and over 14 days in frozen plasma [28, 29]. However, these studies on preanalytical conditions have investigated the expression changes in specific sets of miRNAs, and findings on the effects of different anticoagulants used in blood collection tubes and sample storage conditions on comprehensive miRNA expression profiles are still limited.

To establish preanalytical conditions for obtaining highly reproducible data, we investigated the effects of different anticoagulants and storage conditions on miRNA expression profiles, which could potentially contribute to the variability in miRNA expression levels among samples collected at multiple facilities. MiRNA expression profiles acquired from blood samples using NGS were analyzed according to the following parameters: the library concentration, human genome identification rate, ratio of unique sequences and difference in expression profiles. First, we discovered highly reproducible preanalytical conditions by analyzing miRNAs extracted from healthy volunteer samples. Then, using these preanalytical conditions, we collected samples from cancer patients at multiple independent facilities, measured the miRNA expression level, and evaluated the expression distribution range among facilities.

## Materials and methods

### Blood sample collection from healthy volunteers

Blood samples were collected from 18 healthy volunteers employed by DeNA Co., Ltd. and DeNA Life Science, Inc. Participants were healthy adults who had been examined by a physician and had no diseases. Whole blood was collected by venipuncture into anticoagulant-containing tubes at time of the physical examination. To investigate the effect of whole-blood storage conditions, samples were refrigerated for one hour, six hours, one day, two days and three days after collection. To investigate the effect of storage periods, samples were refrigerated within one hour after collection. To investigate the effects of anticoagulant type, blood samples were collected from each donor using tubes containing EDTA-2Na, EDTA-2K, sodium fluoride and sodium citrate. Plasma separation was performed within two hours after collection. To investigate the effects of storage periods, blood samples were collected using EDTA-2Na tubes. All samples were mixed immediately by inverting the tube 10 times after sample collection. Before participating in this study, the participants completed a written informed consent form. This research was approved by the ethics committee of DeNA Life Science, Inc. (approval number: 20180228_1) and was conducted according to the guidelines of the Declaration of Helsinki.

### Blood sample collection from cancer-free donors in the biobank

Blood samples were obtained from 60 cancer-free donors and stored in the biobank of the National Cancer Center (NCC) Institute for Cancer Control. Cancer-free donors were defined as donors who were not diagnosed with cancer during cancer screening performed by the NCC. Whole blood was collected by venipuncture into EDTA-2Na-containing tubes before treatments. All samples were mixed immediately by inverting the tube 10 times after sample collection. The collected samples were refrigerated within 30 minutes. Before participating in this study, the participants completed a written informed consent form. This research was approved by the NCC Review Board (approval number: 2018–200) and was conducted according to the guidelines of the Declaration of Helsinki.

### Blood sample collection from cancer patients

Blood samples were collected from 60 cancer patients. Whole blood was collected by venipuncture into EDTA-2Na-containing tubes before treatments. All samples were mixed immediately by inverting the tube 10 times after sample collection. The collected samples were stored at 4°C for 30 minutes. From facility A (n = 20), samples from esophageal (11), gastric (5), and pancreatic (4) cancer patients were collected. From facility B (n = 20), samples from esophageal (5), gastric (6), and pancreatic (9) cancer patients were collected. From facility C (n = 20), samples from esophageal (1), gastric (10), and pancreatic (9) cancer patients were collected. All samples were randomly collected without any selection of patients with specific cancer types. Before participating in this study, the participants completed a written informed consent form. This research was approved by Juntendo University Hospital, NCC, and the Osaka International Cancer Center Review Board (approval number: 2020111, 2018–200 and 20043–2) and was conducted according to the guidelines of the Declaration of Helsinki.

### Plasma sample preparation

During the collection of samples from healthy volunteers, the samples were centrifuged once at 1,200 ×g at 4°C for 10 minutes. During the collection of samples from cancer-free patients, the samples were centrifuged once at 1,830 ×g at 4°C for 10 minutes within 12 hours of venipuncture. During the collection of samples from cancer patients, samples were centrifuged once at 1,500 ×g at 4°C for 10 minutes within 12 hours of venipuncture. The supernatant was immediately removed without aspiration of the buffy coat and then cryopreserved in RNase-free cryotubes. There was no significant difference in the amount of miRNA recovered, and the correlation of miRNA profiles was more than 0.99 when the centrifugation conditions were selected between 1,200 x g and 1,900 x g (S1 Fig).

### RNA purification

MiRNAs were automatically extracted from 300 μL of plasma using the Maxwell RSC miRNA Plasma and Serum Kit (Promega, Madison, US) with the Maxprep Liquid Handler (Promega) and Maxwell RSC (Promega) equipment. Purifications were performed according to the manufacturer’s instruction manual. The RNA concentration was measured by a QuantiFluor RNA System (Promega) with a GloMax Explorer (Promega).

### Reverse transcription and sequencing data acquisition

Complementary DNA (cDNA) libraries were synthesized from 5 μL of RNA eluate with 22 polymerase chain reaction (PCR) cycles using the QIAseq miRNA Library Kit (QIAGEN, Venlo, NL) with the Biomek i5 Automated Liquid Handling Workstation (Beckman Coulter, Brea, US). The concentration of each amplification product was measured with a QuantiFluor ONE ds DNA System (Promega). cDNA libraries were merged in one tube at a final concentration of 1.0–1.5 pmol/L. Small RNA sequencing data were obtained using NextSeq 550Dx with the NextSeq 500/550 High Output Kit v2.5, 75 cycles (Illumina, San Diego, US) and the NextSeq Phix Control Kit (Illumina). An automated program was set up for each instrument according to the manufacturer’s instruction manual, and all the samples were processed using the same program.

### MiRNA expression analysis

The raw sequencing data were processed using fastp (version 0.19.6) [30] to remove data regarding the amplification adapter sequence. The unique molecular identifier (UMI) [31] sequences and the probe adapter were trimmed using umi_tools (version 1.0.1) [32]. Mapping was performed using Bowtie (1.3.0), and hg19 was used as the reference human genome. The following options were applied: -n0, -v0, -l18, -m1, and—best. After sorting with SAMtools (version 1.7) [33], an index was created and deduplicated with umi_tools. Then, featureCounts (version 2.0.0) [34] was used to obtain the expression count levels of each miRNA with reference to miRBase (version 20) [1]. To estimate RNA quality from the sequence reads, the UMI ratio was calculated from the total number of UMIs and the total reads obtained from each sample. The human genome identification rate (identification rate) was a number obtained by dividing total number of quality filtered reads processed by fastp (Assigned values) by total number of quality filtered reads processed by fastp (Reads_processed). These parameters are referred to in the column descriptions in S1 Table. MiRNA expression counts are shown in S2 Table.

### Statistical analysis

We calculated the means and standard deviations of the miRNA expression counts, generated scatter plots, and performed one-way analysis of variance (ANOVA) with a post hoc Tukey test or Dunnett’s test using the statistical analysis software R (version 3.6.3) [35]. One-way ANOVA with the Tukey test was applied to compare the miRNA levels during the investigation of the effects of different blood collection tubes among facilities. One-way ANOVA with Dunnett’s test was applied to compare the miRNA levels during the investigation of time-dependent differences. The fold change depicted in the scatter plot was calculated from the miRNA expression counts. The data points on the solid line indicate miRNAs with equal expression levels, and the points above or below the dashed line indicate that miRNAs with expression levels that were 2-fold higher or 1/2-fold lower, respectively.

## Results

### Study design

**Fig 1:**
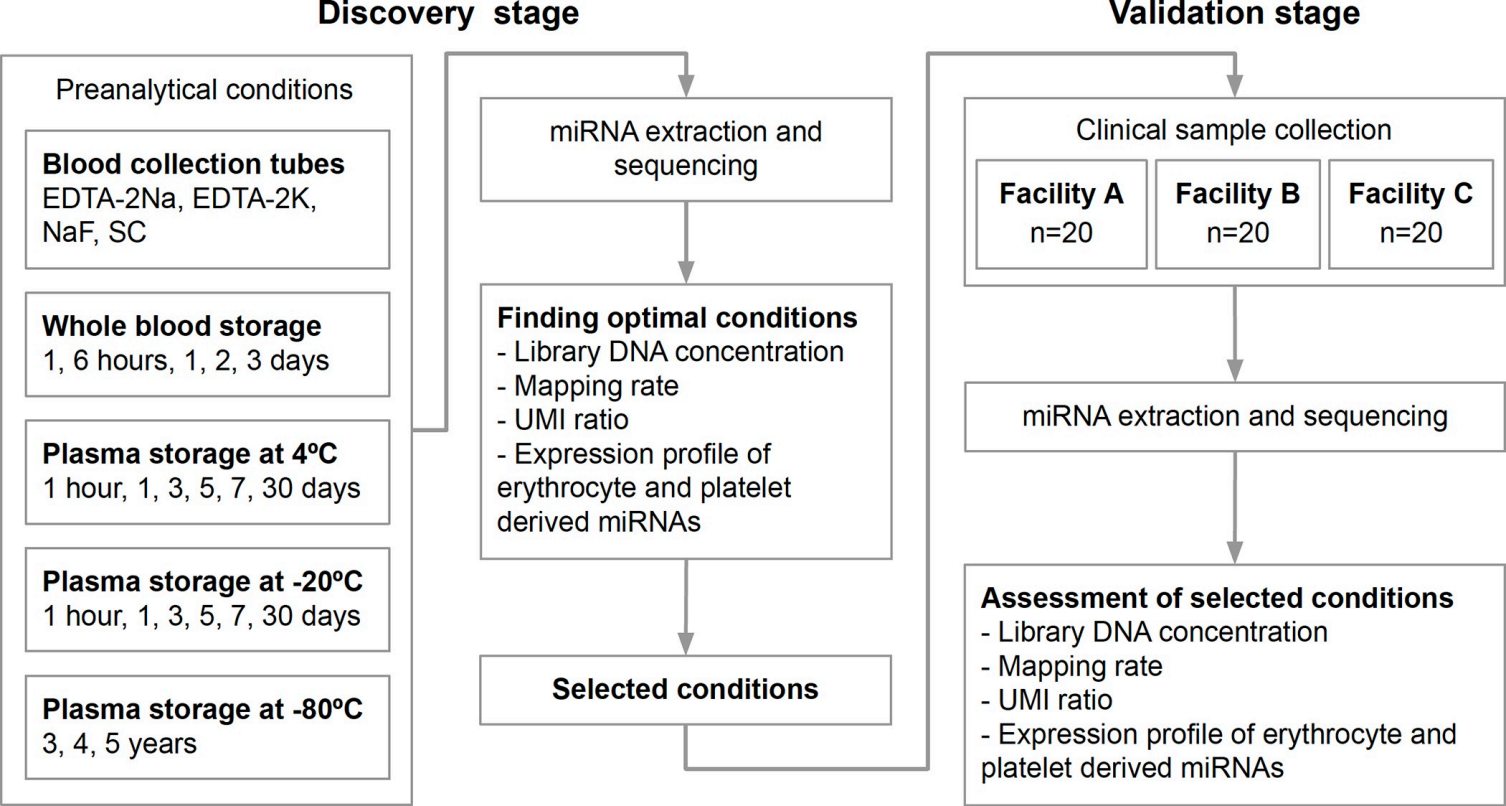
Schematic flow of the discovery and validation of the optimal preanalytical conditions. The effects of different blood collection tubes and sample storage periods were examined during the discovery stage. The library concentrations, identification rate, UMI ratios, and expression profiles were used to identify optimal conditions. During the validation stage, 60 blood samples from cancer patients were collected from three separate facilities to assess the effects of the different conditions. The library concentrations, identification rate, UMI ratios, and expression profiles were assessed to determine whether these measurements were within the assumed range and to validate the preanalytical conditions.

In this study, we explored a standardized protocol for the collection of samples from clinical cancer patients from multiple facilities. First, the preanalytical conditions were investigated after miRNA expression profiles were acquired from plasma samples obtained from healthy volunteers. Then, we evaluated the use of the established preanalytical conditions as a method for collecting samples from clinical cancer patients. The differences between samples subjected to different preanalytical conditions, including different types of blood collection tubes, were evaluated, and the analysis included assessments of the stability of the samples (Fig 1).

During the discovery stage, preanalytical conditions were examined using plasma samples from healthy individuals. During the investigation of the effects of anticoagulants, blood samples from the same donor were collected into EDTA-2Na, EDTA-2K, sodium fluoride (NaF) and sodium citrate (SC) blood collection tubes, which are used in a typical clinical laboratory. The storage stability of refrigerated or frozen samples was evaluated in both whole-blood samples and plasma samples. Whole-blood samples were stored for one hour, six hours (room temperature), one day, two days, and three days at 4°C. Plasma samples were stored at 4°C, -20°C or -80°C. The storage periods used for samples maintained at 4°C and -20°C were one hour, one day, three days, five days, seven days and 30 days. The storage periods for samples maintained at -80°C were three, four and five years. During the discovery stage, we analyzed the following factors to identify the optimal preanalytical conditions. First, the library concentration was measured to examine the effect on biochemical processes, such as RNA extraction and reverse transcription. Second, the identification rate was calculated to examine the effect on the purity of sequences identified as originating from the human genome in the extracted RNA fragments. Third, the UMI ratio was calculated to examine the effect on the uniqueness of the origin RNA located in the library DNA. Finally, the expression levels of erythrocyte-derived miRNAs and platelet-derived miRNAs were compared to examine hemolysis and platelet contamination.

During the validation stage, the selected preanalytical conditions were assessed using 60 samples from clinical cancer patients. Samples were obtained from 20 patients each from facilities A, B and C. The library concentrations, identification rate and UMI ratios were measured. Then, the adaptability of the selected preanalytical conditions was assessed as follows: (i) whether each measurement was significantly different between facilities and (ii) whether measurements obtained from one facility fit within the standard deviation of the results obtained from another facility.

### Evaluation of different blood collection tubes

**Fig 2:**
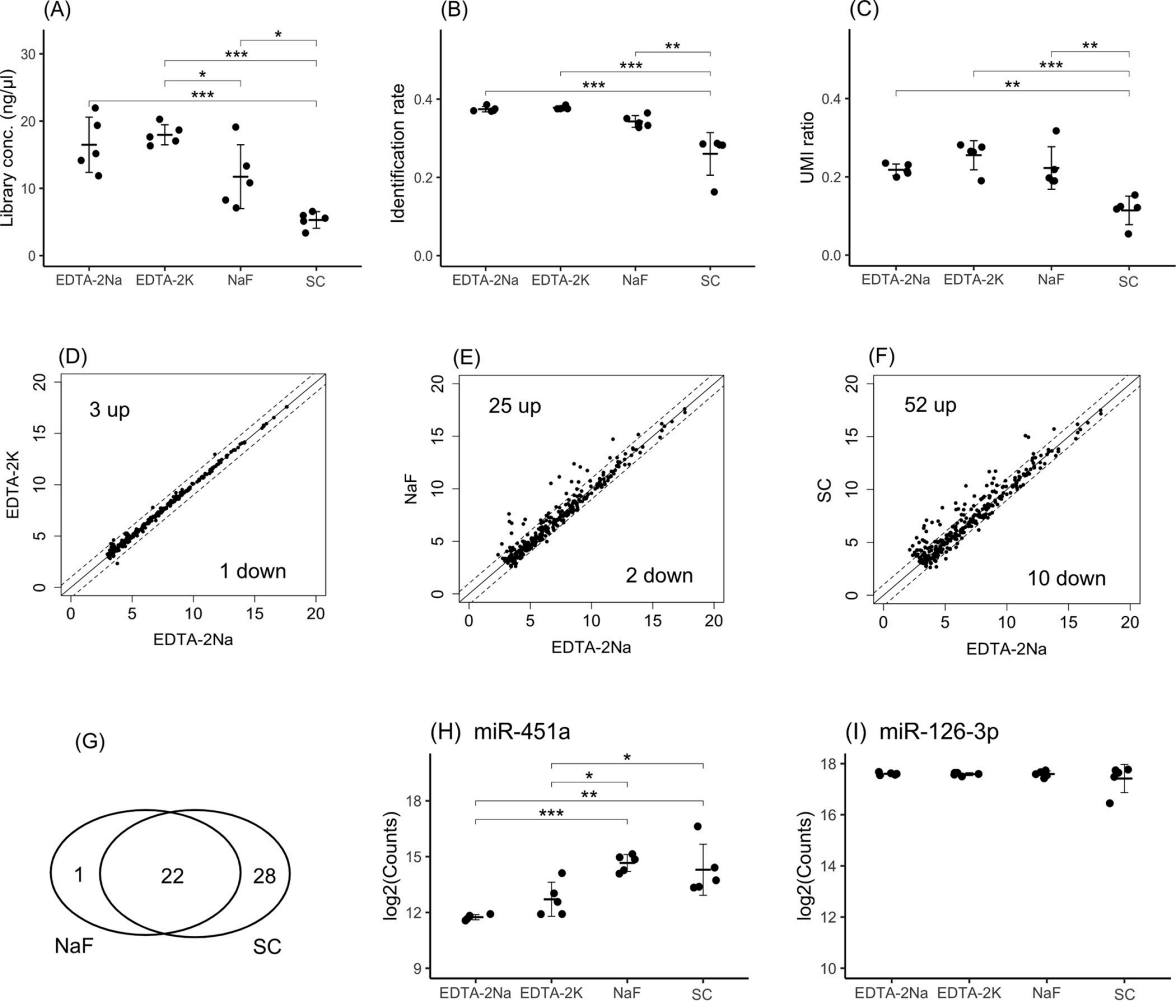
Comparisons among samples obtained in different blood collection tubes. The library concentrations (A), identification rate (B) and UMI ratios (C) were evaluated and compared among samples obtained in EDTA-2Na, EDTA-2K, NaF and citrate blood collection tubes. The mean miRNA expression profiles observed following the use of EDTA-2Na vs. EDTA-2K (D), EDTA-2Na vs. NaF (E) and EDTA-2Na vs. citrate (F) were compared. MiRNAs with levels that exhibited a fold change greater than 2-fold or less than half-fold are indicated outside of the dashed lines. The x-axis or y-axis of the scatter plot indicates log2(miRNA expression counts). The Venn diagram indicates that 24 miRNAs exhibited expression levels that were aligned across all tubes, while the levels of 1 and 28 miRNAs were increased only in NaF or citrate tubes, respectively (G). The expression levels of hsa-miR-451a (H), an indicator of hemolysis, and hsa-miR-126-3p (I), an indicator of platelet contamination, are shown. The crossbar indicates the mean, and the error bar indicates the standard deviation (SD). Significant differences were identified using one-way ANOVA with a post hoc Tukey test and are indicated as * p<0.05, ** p<0.01 and *** p<0.001.

The impacts of anticoagulants were examined using EDTA-2Na, EDTA-2K, NaF and SC tubes. Regarding the library concentration, although there was no significant difference resulting from the use of EDTA-2Na and EDTA-2K tubes, the use of NaF and SC tubes resulted in lower library concentrations than the use of EDTA tubes (Fig 2A). Regarding the identification rate, the use of EDTA-2Na, EDTA-2K and NaF tubes did not result in difference, but the use of SC tubes resulted in a lower identification rate (Fig 2B). Regarding the UMI ratio, the use of EDTA-2K tubes resulted in the highest UMI ratios, but the UMI ratios did not differ following the use of EDTA-2Na and NaF tubes. There were significant differences resulting from the use of EDTA tubes and SC tubes (Fig 2C). It was observed that EDTA-2K and EDTA-2Na exhibited similar properties, while NaF and SC exhibited different properties from EDTA.

To determine the differences in the expression of miRNAs resulting from the use of various blood collection tubes, a profile comparison was performed in three ways: EDTA-2Na vs. EDTA-2K tubes (Fig 2D), EDTA-2Na vs. NaF tubes (Fig 2E) and EDTA-2Na vs. SC tubes (Fig 2F). The numbers of miRNAs that exhibited increased levels of more than 2-fold between samples obtained in EDTA-2Na and EDTA-2K tubes, EDTA-2Na and NaF tubes and EDTA-2Na and SC tubes were 2, 25, and 52, respectively. The numbers of miRNAs that exhibited decreased levels of less than 1/2-fold between samples obtained in EDTA-2Na and EDTA-2K tubes, EDTA-2Na and NaF tubes and EDTA-2Na and SC tubes were 1, 2, and 10, respectively. No common features were observed among the miRNAs with decreased expression levels. The miRNAs that exhibited increased expression in samples obtained in NaF and SC tubes were examined, and 24 miRNAs were identified (Fig 2G). Table 1 shows the list of these miRNAs ranked by their expression levels. Hsa-let-7b-5p [36], hsa-miR-451a [37], hsa-miR-144 [38], hsa-miR-486 [39], hsa-miR-363 [40], hsa-miR-96 [41] and hsa-miR-4732-5p [36] have been reported as erythrocyte-derived miRNAs. The fold change in the levels of erythrocyte-derived hsa-miR-451a between samples obtained from EDTA-2Na and those obtained from NaF tubes and EDTA-2Na and SC tubes was more than 6-fold (Table 1), with significant differences between samples obtained from EDTA-2Na and NaF tubes or SC tubes (Fig 2H). In addition, the expression levels of platelet-derived hsa-miR-126-3p were compared to determine the degree of platelet contamination, but a significant difference was not observed (Fig 2I). In this study, we focused on hsa-miR-126-3p as a representative marker to investigate the expression of platelet-derived miRNAs [42]. The expression levels of other platelet-derived miRNAs [43, 44], such as hsa-miR-223-3p, hsa-let-7f-5p and hsa-miR-16-5p, were investigated, and there were no differences in expression between samples obtained in the different tubes (S2 Fig). To examine differences in RNA concentrations, blood samples were collected from five donors in EDTA-2Na, EDTA-2K, NaF, and citrate blood collection tubes. Samples obtained in EDTA-2Na and EDTA-2K tubes exhibited the highest RNA concentrations, which were not significantly different among them, but the RNA concentrations in samples obtained from NaF and citrate samples were lower (S3A Fig). In addition, differences among samples subjected to different storage periods were examined. Blood samples were collected from five donors in EDTA tubes at 1 hour, 6 hours (RT), 1 day, 2 days, and 3 days at 4°C. There was no significant difference in the concentration of RNA between samples extracted at 1 hour after collection and those collected at each other time point (S3B Fig).

**Table 1:** MiRNAs with higher expression in NaF and SC than in EDTA-2Na.

	Mean of normalized counts (log2)		
	NaF	SC	EDTA-2Na
let-7b-5p	15.1	15.6	13.8
miR-451a	14.7	14.3	11.7
miR-122-5p	12.7	14.3	11.4
miR-144-3p	12.3	11.3	9.0
miR-182-5p	11.9	11.3	8.9
miR-144-5p	11.1	10.8	8.7
miR-363-3p	10.7	10.5	8.5
miR-192-5p	10.6	10.1	8.4
miR-183-5p	10.3	9.7	7.9
miR-150-5p	9.9	9.0	7.2
miR-3613-5p	9.5	8.9	7.1
miR-20b-5p	8.3	8.5	6.6
miR-3615	7.6	7.9	6.5
miR-96-5p	7.5	7.9	6.3
miR-106b-5p	7.5	7.4	5.7
miR-16-2-3p	7.1	6.7	4.5
miR-375	6.9	6.6	4.4
miR-1180-3p	6.9	6.2	3.4
miR-4732-5p	6.6	6.1	3.4
miR-486-5p	6.6	5.9	3.3
miR-1294	5.7	5.5	3.2
miR-92b-5p	4.7	5.2	3.1

From the results of the differences in samples obtained from different blood collection tubes, it was observed that EDTA-2Na and EDTA-2K tubes had similar properties. However, the properties of NaF and SC tubes were different from those of EDTA tubes, suggesting that hemolysis was promoted in these tubes.

### Stability of whole blood over storage periods of up to three days at 4°C

**Fig 3:**
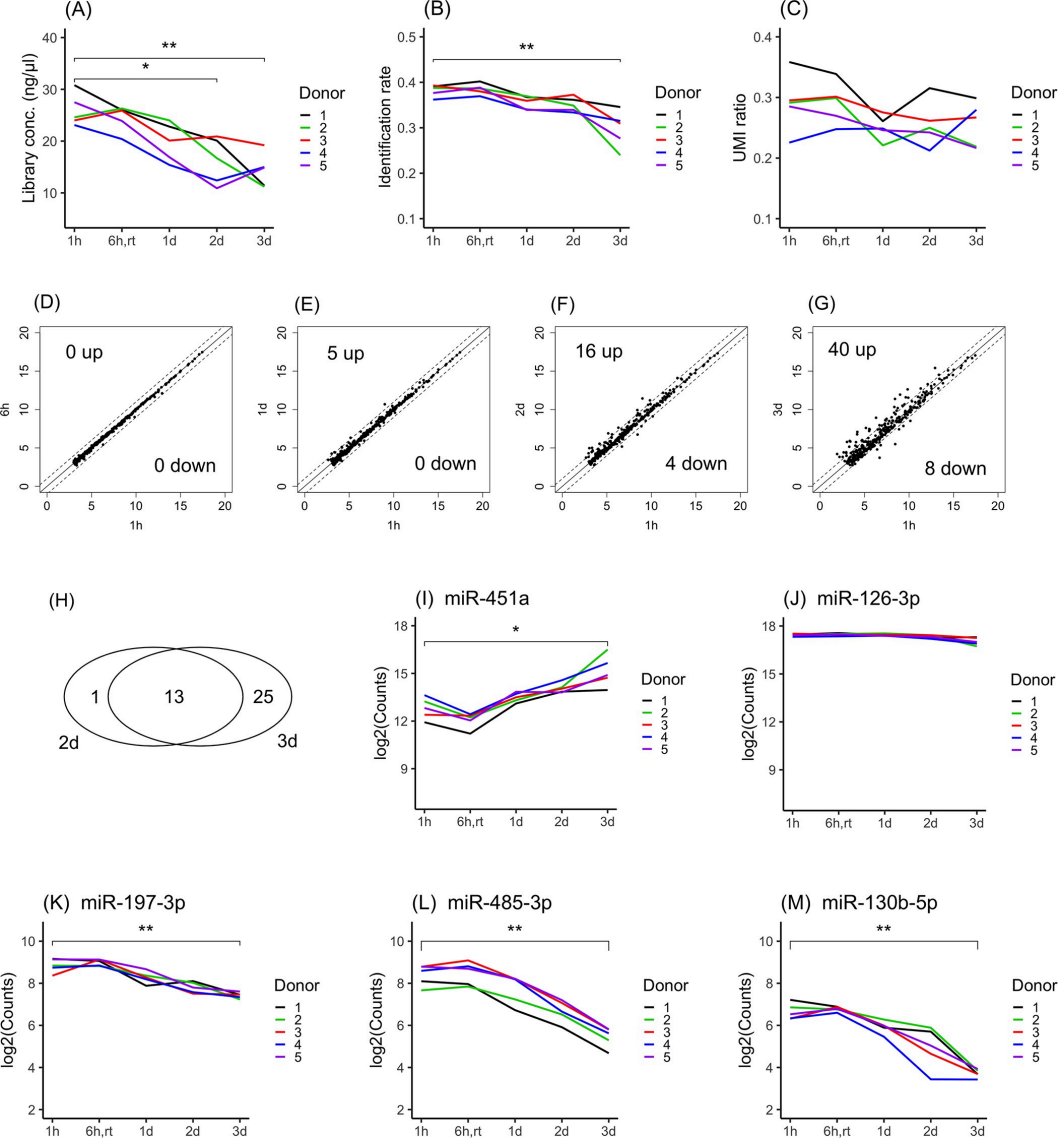
Stability of whole blood in storage periods up to three days at 4°C. The library concentrations (A), identification rate (B) and UMI ratios (C) were compared between samples stored for 1 hour and 6 hours (room temperature) and 1 day, 2 days and 3 days after blood collection. The mean miRNA expression profiles at 1 hour vs. 6 hours (D), 1 hour vs. 1 day (E), 1 hour vs. 2 days (F) and 1 hour vs. 3 days (G) were compared. MiRNAs with levels that exhibited a fold change greater than 2-fold or less than half-fold are indicated outside of the dashed lines. The X-axis or Y-axis of the scatter plot indicates log2(miRNA expression counts). The Venn diagram indicates that the levels of 15 miRNAs were aligned across samples obtained from all tubes, while 1 and 25 miRNAs exhibited increased levels only at 2 days or 3 days, respectively (H). The expression levels of hsa-miR-451a (I), an indicator of hemolysis, and hsa-miR-126-3p (J) and an indicator of platelet contamination are shown. To illustrate the level of sample degradation in a time-dependent manner, the levels of hsa-miR-197-3p (K), hsa-miR-485-3p (L) and hsa-miR-130b-5p (M) are shown. Significant differences were identified using one-way ANOVA with a post hoc Dunnett’s test and are indicated as * p<0.05, ** p<0.01.

The storage stability of whole-blood samples was investigated. To examine the time-dependent stability of samples subjected to various storage periods, blood samples stored for one hour after blood collection were used as the baseline condition and compared with blood samples stored for 1 day, 2 days, and 3 days. Plasma samples were prepared after these storage periods, and various measurements were performed. In addition, plasma samples prepared after 6 hours of storage at room temperature were examined for reference information. The library concentration was decreased in a time-dependent manner in all donor samples. Significant differences were observed after one day (Fig 3A). The identification rate was decreased in a time-dependent manner in all donor samples. Significant differences were observed after two days (Fig 3B). However, the UMI ratio showed no time-dependent differences in any donor samples (Fig 3C). From these results, it was observed that the quality of whole-blood samples did not change until one day after blood collection.

To investigate the time-dependent change in miRNA expression, miRNA expression profiles were compared between samples stored for one hour and samples stored for various periods. The numbers of miRNAs with levels that increased more than 2-fold between one hour and six hours, one day, two days, and three days were 0, 5, 14, and 38, respectively (Fig 3D–3G). Thirteen of the 38 miRNAs that exhibited levels that were increased in samples stored for three days were also found to have increased levels in samples stored for two days (Fig 3H). Table 2 shows the mean expression levels of these 13 miRNAs. The miRNA with the highest expression was hsa-miR-451a, followed by hsa-miR-144-3p. These miRNAs have been reported as erythrocyte-derived miRNAs. The levels of hsa-miR-451a and hsa-miR-144-3p were found to increase by more than five- and sixfold, respectively, from one hour to three days (Table 2). The expression level of hsa-miR-451a significantly increased from one hour to 3 days of storage (Fig 3I). However, no significant difference was observed in the levels of hsa-miR-126-3p, suggesting that platelet destruction did not progress during storage (Fig 3J). The expression levels of the platelet-derived miRNAs hsa-223-3p, hsa-let-7f-5p and hsa-miR-16-5p did not differ during the storage periods (S4 Fig). In addition, to examine markers reflecting sample degradation, miRNA expression profiles in samples stored for each storage period were compared with miRNA expression profiles in samples stored for one hour after blood collection. The numbers of miRNAs with expression levels that decreased less than 1/2-fold between one hour and 6 hours, 1 day, 2 days, and 3 days were 0, 0, 4, and 8, respectively. The expression levels of hsa-miR-197-3p (Fig 3K), hsa-miR-485-3p (Fig 3L), and hsa-miR-130b-3p (Fig 3M) were observed to decrease in a time-dependent manner. It was suggested that these miRNAs could be useful as markers for monitoring the degradation of whole-blood samples.

**Table 2:** MiRNAs with higher expression at 2 days and 3 days than at 1 hour.

	Mean normalized counts (log2)		
	1 hour	2 days	3 days
miR-451a	12.9	14.1	15.4
miR-144-3p	9.8	11.2	12.6
miR-150-5p	9.0	11.4	11.9
miR-144-5p	8.4	9.9	11.4
miR-183-5p	6.3	7.6	8.9
miR-483-5p	5.2	6.9	7.6
miR-193a-5p	4.2	5.5	6.8
miR-99a-5p	5.6	6.8	7.4
miR-4732-5p	3.4	4.7	5.8
miR-96-5p	4.2	5.5	7.1
miR-1180-3p	3.0	5.0	5.8
miR-205-5p	3.3	5.0	5.9
miR-1294	2.7	3.7	4.4

We found that hemolysis gradually increased during the storage period, and miRNAs that could be used for monitoring blood degradation were identified. From the results described above, it was found that the quality of whole-blood samples could be maintained if samples were stored within 6 hours at room temperature and within one day at 4°C.

### Stability of plasma over storage periods of up to 30 days at 4°C

**Fig 4:**
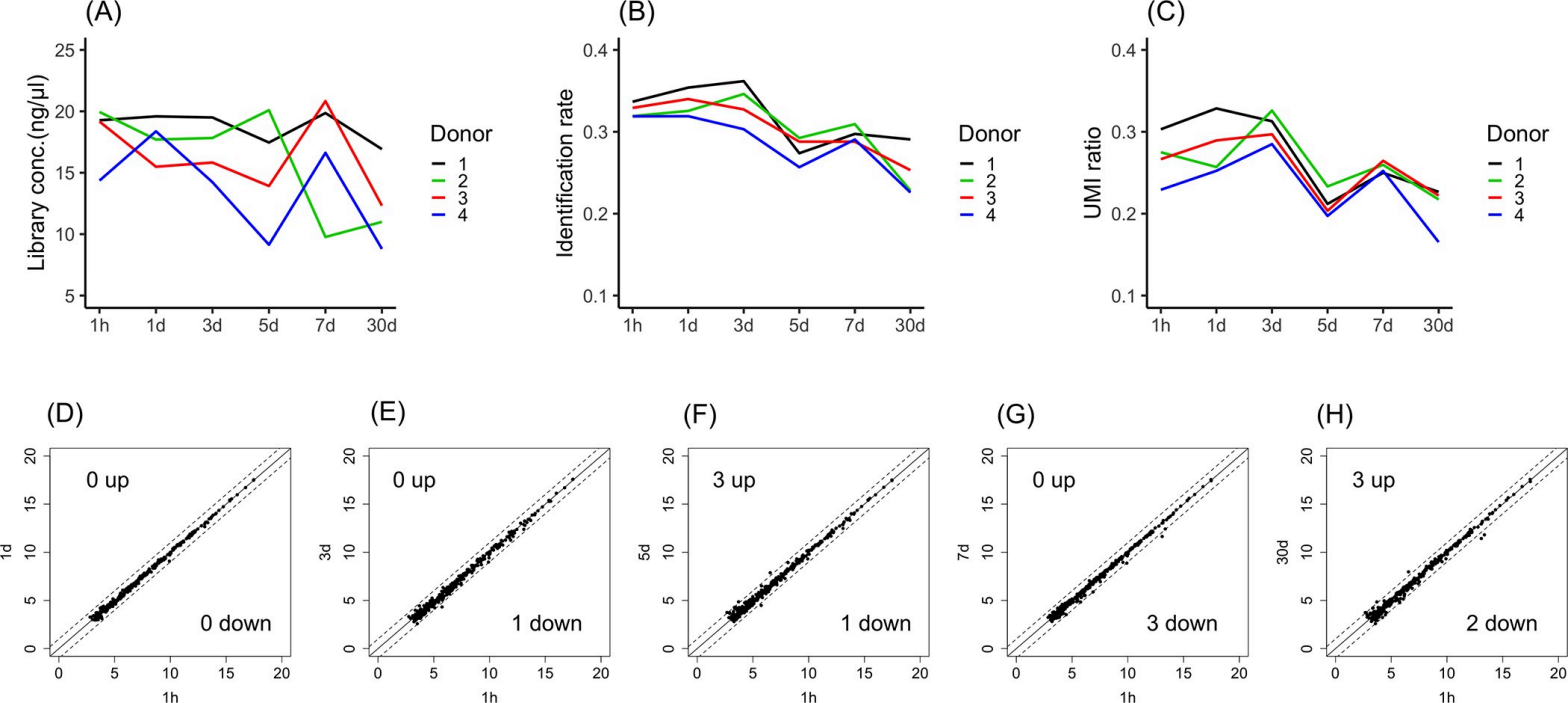
Stability of plasma in storage periods up to 30 days at 4°C. The library concentrations (A), identification rate (B) and UMI ratios (C) were compared between samples stored for 1 hour and 1 day, 3 days, 5 days, 7 days and 30 days after blood collection. MiRNA expression profiles at 1 hour vs. 1 day (D), 1 hour vs. 3 days (E), 1 hour vs. 5 days (F), 1 hour vs. 7 days (G), and 1 hour vs. 30 days (H) were compared. MiRNAs with a fold change greater than 2-fold or less than half-fold are indicated outside of the dashed lines. The X-axis or Y-axis of the scatter plot indicates log2(miRNA expression counts). Significant differences were identified using one-way ANOVA with a post hoc Dunnett’s test.

Depending on the laboratory conditions, plasma samples are sometimes preserved in cold storage before freezer storage. Thus, the stability of plasma samples stored at 4°C was investigated. To examine the time-dependent stability of samples during the storage period, plasma samples stored for one hour after separation from whole blood were used as the baseline condition and compared with samples stored for 1 day, 3 days, 5 days, 7 days and 30 days. Although the library concentration (Fig 4A), identification rate (Fig 4B) and UMI ratio (Fig 4C) decreased in a time-dependent manner in all donor samples, significant differences among samples stored for the various storage periods were not observed.

To investigate the time-dependent change in expression, miRNA expression profiles were compared between samples subjected to one hour of storage and samples stored for other periods. The numbers of miRNAs with levels that increased more than 2-fold between samples stored for one hour and those stored for one day, three days, five days, seven days and 30 days were 0, 0, 3, 0 and 3, respectively (Fig 4D–4H). However, the numbers of miRNAs with levels that decreased less than 1/2-fold between one hour and one day, three days, five days, seven days and 30 days were 0, 1, 1, 3 and 2, respectively (Fig 4D–4H). No common features were observed in either the miRNAs with increased expression miRNAs or those with decreased expression. Overall, the miRNA expression profiles did not show any remarkable changes after 30 days at 4°C.

### Stability of plasma over storage periods of up to 30 days at -20°C

**Fig 5:**
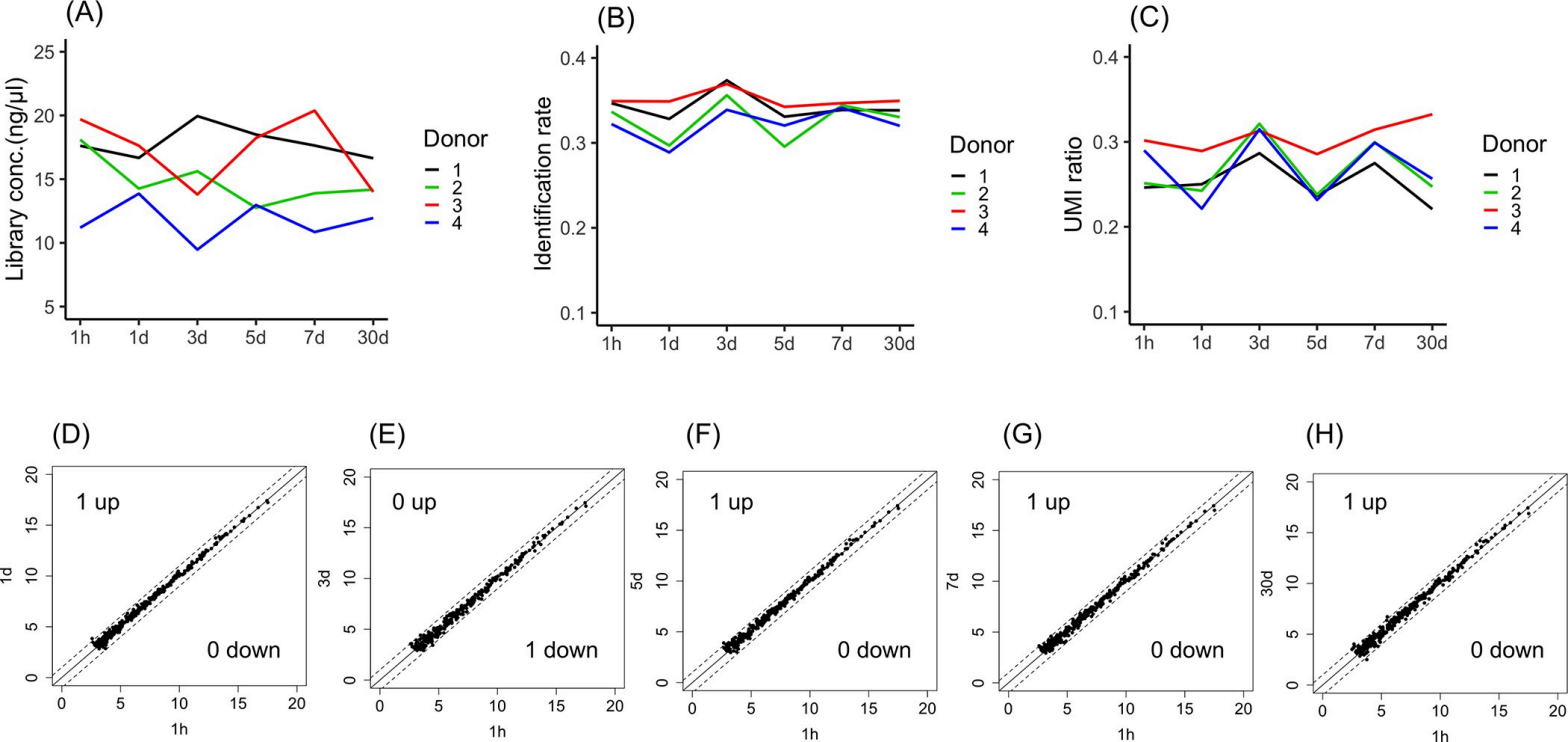
Stability of plasma in storage periods up to 30 days at -20°C. The library concentrations (A), identification rate (B) and UMI ratios (C) were compared between samples stored for 1 hour and 1 day, 3 days, 5 days, 7 days and 30 days after blood collection from five healthy volunteers. The mean miRNA expression profiles of samples stored for 1 hour vs. 1 day (D), 1 hour vs. 3 days (E), 1 hour vs. 5 days (F), 1 hour vs. 7 days (G), and 1 hour vs. 30 days (H) were compared. MiRNAs with levels that exhibited a fold change greater than 2-fold or less than half-fold are indicated outside of the dashed lines. The X-axis or Y-axis of the scatter plot indicates log2(miRNA expression counts). Significant differences were identified using one-way ANOVA with a post hoc Dunnett’s test.

Plasma samples are routinely stored in a freezer until measurement experiments. Thus, the stability of plasma samples at -20°C was investigated. To examine the time-dependent stability of samples during the storage period, plasma samples stored for one hour after separation from whole blood were used as the baseline condition and compared with samples stored for 1 day, 3 days, 5 days, 7 days and 30 days. The library concentration (Fig 5A), identification rate (Fig 5B) and UMI ratio (Fig 5C) did not significantly differ among samples subjected to the various storage periods.

To investigate the time-dependent change in expression, miRNA expression profiles were compared between samples subjected to one hour of storage and samples stored for other periods. The numbers of miRNAs with levels that increased more than 2-fold between one hour and one day, three days, five days, seven days and 30 days were 1, 0, 1, 1 and 1, respectively (Fig 5D–5H). However, the numbers of miRNAs with levels that decreased less than 1/2-fold between one hour and one day, three days, five days, seven days and 30 days were 0, 1, 0, 1 and 1, respectively (Fig 5D–5H). No common features were observed among miRNAs with increased expression and those with decreased expression. Overall, the miRNA expression profiles did not show any remarkable changes after 30 days at -20°C.

### Stability of plasma samples from a biobank over storage periods of up to five years at -80°C

**Fig 6:**
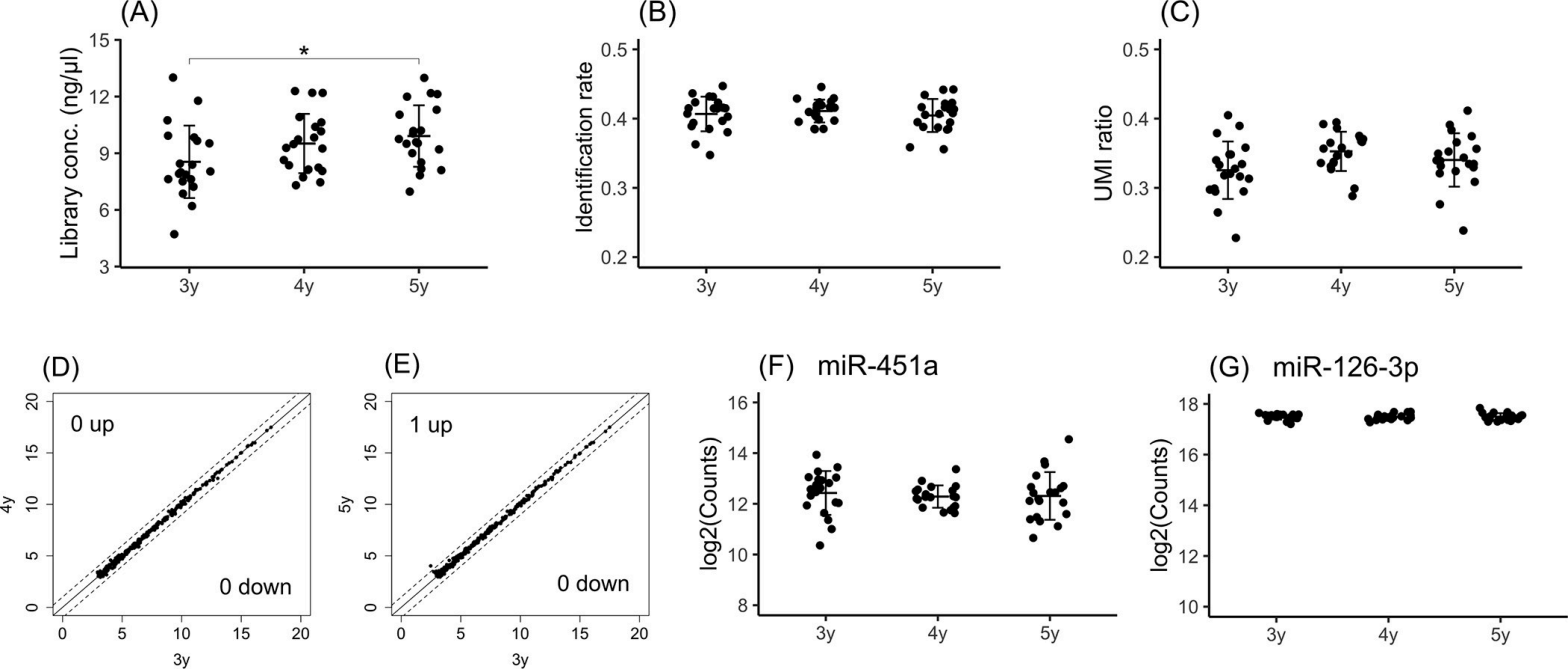
Stability of plasma samples from a biobank over storage periods of up to five years. The library concentrations (A), identification rate (B) and UMI ratios (C) were compared among samples stored for three years, four years and five years. The miRNA expression profiles of samples stored for 3 years vs. 4 years (D) and 3 years vs. 5 years (E) were compared. MiRNAs with levels that exhibited a fold change greater than 2-fold or less than a half-fold are indicated outside of the dashed lines. The X-axis or Y-axis of the scatter plot indicate log2(miRNA expression counts). The expression levels of hsa-miR-451a (F), an indicator of hemolysis, and hsa-miR-126-3p (G), an indicator of platelet contamination, are shown. The crossbar indicates the mean, and the error bar indicates the standard deviation (SD). Significant differences were identified using one-way ANOVA with a post hoc Dunnett’s test and are indicated as * p<0.05.

Samples that have been stored for years in a biobank at -80°C are often used in the development of diagnostic tests using clinical samples. Thus, we investigated the stability of plasma samples obtained from a biobank. To examine the time-dependent degradation of samples over various storage periods, plasma samples stored for 3 years were used as the baseline condition and compared with samples stored for four and five years. The library concentration did not differ between samples stored for 3 years and samples stored for 4 years, but a significant difference was observed between samples stored for 3 years and samples stored for 5 years (Fig 6A). The identification rate (Fig 6B) and UMI ratio (Fig 6C) did not significantly differ among samples subjected to the various storage periods.

To investigate the time-dependent change in miRNA expression, miRNA expression profiles were compared between samples stored for three years and samples stored for four and five years. The numbers of miRNAs with levels that increased more than 2-fold between three years and after four years and five years were 0 and 1, respectively (Fig 6D and 6E). However, the numbers of miRNAs with levels that decreased less than 1/2-fold between three years and after four years and five years were 0 and 0, respectively (Fig 6D and 6E). Although a significant difference was shown in the library concentration between samples stored for 3 years and samples stored for 5 years, the miRNA expression profiles differed only slightly between samples stored for these periods. To examine the differences in the levels of hemolysis that occurred over different storage periods, the expression of hsa-miR-451a was measured and showed no significant differences between periods (Fig 6F). During the examination of platelet contamination, the expression of hsa-miR-126-3p was measured, but no significant differences in expression were observed between periods (Fig 6G). It was observed that the quality of the 5-year-old samples was maintained. From these results, we found that remarkable quality degradation was not observed in frozen samples stored for years in the biobank.

### Assessment of selected preanalytical conditions using clinical cancer samples

**Fig 7:**
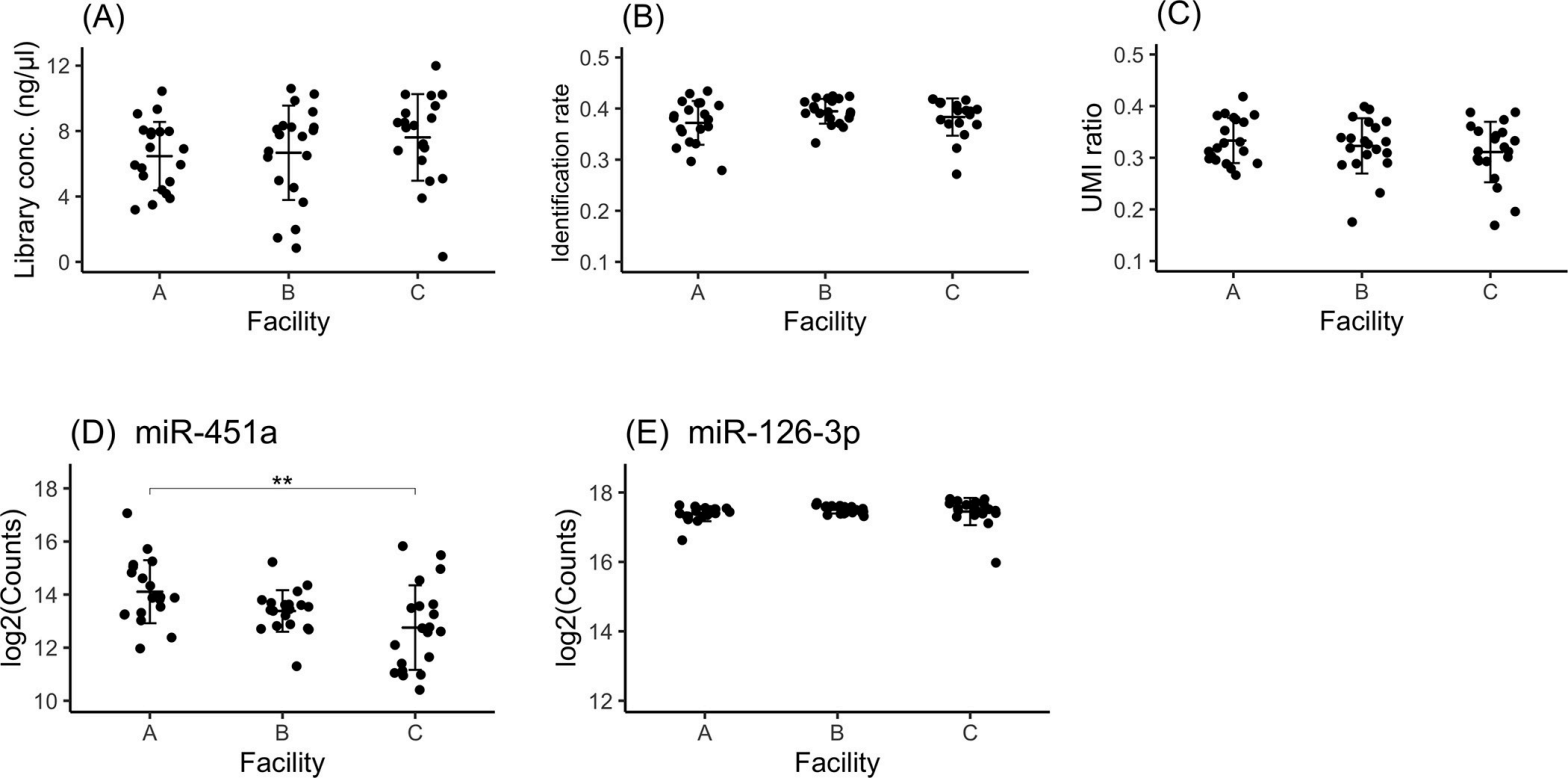
Assessment of estimated conditions using clinical samples from facilities A, B and C. The library concentrations (A), identification rate (B) and UMI ratios (C) were compared among samples obtained from different facilities. The expression levels of hsa-miR-451a (D), an indicator of hemolysis, and hsa-miR-126-3p (E), an indicator of platelet contamination, are shown. The crossbar indicates the mean, and the error bar indicates the standard deviation (SD). Significant differences were identified using one-way ANOVA with a post hoc Tukey test and are indicated as ** p<0.01.

We selected the preanalytical conditions based on the results obtained using healthy samples as follows. Blood samples collected using EDTA blood collection tubes were refrigerated within 1 hour, plasma separation was performed within 12 hours, and the obtained plasma samples were frozen at -80°C. Prior to the large-scale collection of clinical samples, the developed preanalytical conditions were validated using clinical samples collected on a small scale. For this purpose, a total of 60 cancer samples were collected from three facilities (20 samples per facility). First, the samples obtained from cancer patients were evaluated for equivalence among the three facilities in terms of the library concentration (Fig 7A), identification rate (Fig 7B) and UMI ratio (Fig 7C). Significant differences were not observed in these evaluations among facilities. This finding suggested that the properties of the samples were equivalent among different facilities.

Next, to examine the differences in the levels of hemolysis in samples obtained from different facilities, the expression of hsa-miR-451a was compared. The fold changes in the levels of this miRNA in samples collected at facility A vs. facility B, samples collected at facility B vs. facility C and samples collected at facility A vs. facility C were 1.8-fold, 1.2-fold and 2.3-fold, respectively. Significant differences were observed between samples obtained in facilities A and C. However, the mean expression of hsa-miR-451a in samples collected at facility A was within a standard deviation of the mean expression of hsa-miR-451a in samples collected at facilities B and C. The same was true in comparisons of samples obtained from each individual facility with samples obtained from the other two (Fig 7D). The variation in hemolysis levels between samples obtained from different facilities did not exceed the variation observed in samples obtained within facilities. In addition, to evaluate the differences in the levels of platelet contamination that occurred in samples obtained from different facilities, hsa-miR-126-3p expression was measured. There were no significant differences in hsa-miR-126-3p expression among samples obtained from facilities A, B and C (Fig 7E). The expression levels of platelet-derived miRNAs hsa-miR-223-3p, hsa-let-7f-5p and hsa-miR-16-5p were not different among samples obtained from the different facilities (S5 Fig).

From these assessments it was revealed that major differences in the library concentration, identification rate, UMI ratio or platelet contamination were not observed among samples obtained from the three facilities. Although significant differences in hemolysis levels were observed among samples obtained from the different facilities, those differences were not based on the specific features of the facilities. The selected conditions were thought to demonstrate robustness in the sample collection methods used among facilities. We found that the optimal preanalytical conditions were established for the collection of samples from cancer patients in multiple facilities.

## Discussion

To determine the optimal preanalytical conditions for acquiring highly reproducible data, we investigated the effects of anticoagulants and sample storage conditions on miRNA expression profiles using plasma samples from healthy volunteer. Variances in the miRNA concentration, library concentration, human genome identification rate, ratio of unique sequences and expression profiles were compared between samples subjected to each condition. Then, the optimal preanalytical conditions for acquiring highly reproducible data were established. The selected preanalytical conditions included the use of EDTA as the anticoagulant, whole blood storage at room temperature for 6 hours or 4°C for 24 hours, and plasma storage at 4°C or -20°C for within 30 days or -80°C for within 5 years. Next, a protocol was formulated with these preanalytical conditions, and samples obtained from cancer patients were collected from three independent facilities. We found no significant differences in the distribution range of measurements among samples obtained from the different facilities. The preanalytical conditions identified in this study were shown to be practical for developing protocols to collect samples from multiple facilities.

Plasma and serum are used at approximately the same frequency for cancer liquid biopsy. A review article of studies on cancer-specific miRNAs reported that of 154 cancer-specific miRNAs, 42% were discovered using plasma, 57% using serum, and 1% using both [23]. To our knowledge, there is no consensus on whether plasma or serum should be used in discovery studies for cancer-specific miRNA markers. Regarding serum preparation, differences in miRNA expression profiles between samples obtained from different operators are assumed since there are factors that depend on the manipulation technique, such as the times used for clot formation and the intensity of inversion mixing. However, regarding plasma preparation, there are thought to be fewer technique-dependent variations because only centrifugation is needed after blood collection to obtain plasma samples. For these reasons, we selected plasma so that a robust measurement system could be established by minimizing factors that depend on manual procedures when samples are collected across multiple facilities.

We investigated time-dependent differences in miRNA expression profiles when whole blood was refrigerated for 3 days, and we found that the expression of erythrocyte-derived miRNA (hsa-miR-451a) increased proportionately in a time-dependent manner (Fig 3I). In contrast, the levels of hsa-miR-197-3p (Fig 3K), hsa-miR-485-3p (Fig 3L) and hsa-miR-130b-5b (Fig 3M) were found to decrease in a time-dependent manner. Regarding the storage of plasma samples, it is known that the expression levels of hsa-miR-16 and hsa-miR-21 decrease in a time-dependent manner to approximately 60% and 70% over 100 days at 4°C [28]. Regarding the storage of serum samples, it was reported that miRNA concentrations showed only a slight difference between 2 and 4 years of storage at -20°C, whereas miRNA concentrations decreased to approximately 1/2 after 6 years and to 1/4 after 10 years [45]. However, to the best of our knowledge, there are no miRNAs that exhibited a time-dependent decrease in expression in whole-blood samples, and this is the first report showing that hsa-miR-197-3p, hsa-miR-485-3p and hsa-miR-130b-5b are potential markers for tracking sample degradation in whole blood.

The expression of erythroid-derived hsa-miR-451a was more than 2-fold higher in plasma obtained from NaF and SC tubes than in plasma obtained from EDTA tubes (Fig 2H), and 7 of the 25 miRNAs that were highly expressed in plasma from both NaF and SC tubes were erythroid-derived (Table 1). The degree of hemolysis was higher when blood was collected in NaF or SC blood collection tubes than in EDTA blood collection tubes. We speculated the following on the basis of these results. It has been reported that NaF reduces the amount of linolenic acid, a component of erythrocyte cell membranes, that is incorporated into the cell membrane to 1/3 or less [46], suggesting that intracellular miRNA leaks from the damaged cell membrane compartment. Although we could not find reports on the effects of SC on erythrocyte membranes, it was suspected that if the SC blood collection tubes contain 3.2% SC pH 5.5 solution, which is equivalent to 1/10 the volume of blood, the erythrocyte membrane could be damaged by the pH gradient when the first drops of blood mix with the SC. Therefore, we speculated that plasma collected in NaF and SC tubes would have higher levels of hemolysis than plasma collected in EDTA tubes. Considering the results of this study, which showed higher expression of several erythroid-derived miRNAs in plasma collected from NaF and SC tubes and the effects of NaF and SC on erythrocyte cell membranes, we recommend the use of EDTA as an anticoagulant to reduce hemolysis levels.

The analysis of the expression of erythrocyte-derived hsa-miRNA-451a across facilities revealed that the expression distribution range among facilities was less than the expression distribution range among samples obtained at the same facility (Fig 7D). These results demonstrate the potential for standardizing preanalytical conditions across facilities to establish a robust miRNA assay system. However, significant differences were observed between samples obtained from facility A and facility C (Fig 7D). This finding also suggests that samples obtained from the same facility could show significantly different degrees of hemolysis among individuals. Therefore, when samples are collected from a number of facilities, it is assumed that a certain number of samples with a high degree of hemolysis could be included. A method to standardize the hemolysis level using the differential expression of hsa-miR-451a and hsa-miR-23a-3p has also been reported [37]. To generate a classification model for multiple cancer types, precise miRNA expression profiles are needed. We speculate that these miRNAs could be applied as a quality criterion for data acquisition in the future.

We examined the effect of the type of anticoagulant used for plasma sample preparation and the conditions of sample storage on the comprehensive miRNA expression profile. We concluded the following: (i) EDTA should be used as an anticoagulant in blood collection tubes; (ii) whole blood should be left at room temperature prior to plasma preparation within 6 hours and (iii) refrigerated within 24 hours; and plasma should be stored at (iv) 4°C or -20°C within 30 days and at (v) -80°C within 5 years. These findings are worth considering during determinations of the type of anticoagulant and storage conditions for samples because they contribute to reducing the variation in sample quality among facilities.

## Supporting information

S1 Fig Comparison of expression profiles among samples subjected to different centrifugation conditions. RNA concentration in samples obtained using different centrifugation conditions. Plasma was separated at 1,200 x g, 1,900 x g and 2,800 x g (A). Significant differences were identified using one-way ANOVA with a post hoc Tukey test and are indicated as * p<0.05, ** p<0.01 and *** p<0.001. Analysis of the correlation between miRNA expression levels in plasma. Plasma was separated at 1,200 x g, 1,900 x g and 2,800 x g. The expression levels are expressed as the mean of five different donor samples. MiRNAs with more than 8 TPM were selected and analyzed (B).
(TIFF)

S2 Fig Comparison of platelet-derived miRNA expression among samples collected in different tubes. Hsa-miR-223-3p (A), hsa-let-7f-5p (B) and hsa-miR-16-5p (C) levels were evaluated in samples obtained in EDTA-2Na, EDTA-2K, NaF and citrate tubes. The crossbar indicates the mean, and the error bar indicates the standard deviation (SD). Significant differences were identified using one-way ANOVA with a post hoc Tukey test and are indicated as * p<0.05, ** p<0.01 and *** p<0.001.
(TIFF)

S3 Fig RNA concentrations in the purified RNA samples. Concentration of RNAs obtained from samples isolated using different blood collection tube conditions (A). The crossbar indicates the mean, and the error bar indicates the standard deviation (SD). Significant differences were identified using one-way ANOVA with a post hoc Tukey test and are indicated as * p<0.05, ** p<0.01 and *** p<0.001. Time-dependent changes in RNA concentrations in whole blood stored at 4°C (B). Significant differences were identified using one-way ANOVA with a post hoc Dunnett’s test and are indicated as * p<0.05, ** p<0.01 and *** p<0.001.
(TIFF)

S4 Fig Stability of platelet-derived miRNAs in storage periods up to three days at 4°C. Hsa-miR-223-3p (A), hsa-let-7f-5p (B) and hsa-miR-16-5p (C) levels were evaluated at different times after blood collection: 1 hour, 6 hours (room temperature), 1 day, 2 days and 3 days. Significant differences were identified using one-way ANOVA with a post hoc Dunnett’s test and are indicated as * p<0.05, ** p<0.01 and *** p<0.001.
(TIFF)

S5 Fig Comparison of platelet-derived miRNAs using clinical samples from facilities A, B and C. Hsa-miR-223-3p (A), hsa-let-7f-5p (B) and hsa-miR-16-5p (C) levels were compared among facilities. Significant differences were identified using one-way ANOVA with a post hoc Tukey test and are indicated as * p<0.05, ** p<0.01 and *** p<0.001.
(TIFF)

S1 Table QC data from NGS measurement for comparison of variances among samples subjected to different conditions. (XLSX)

S2 Table Count data of miRNA expression for analysis of miRNA profiles among samples subjected to different conditions. (XLSX)
