# Supplementary figures and images for: Establishment of preanalytical conditions for microRNA profile analysis of clinical plasma samples
Source: PLoS One. 2022 Dec 14;17(12):e0278927. doi: 10.1371/journal.pone.0278927 (PMC9750036; doi:10.1371/journal.pone.0278927)

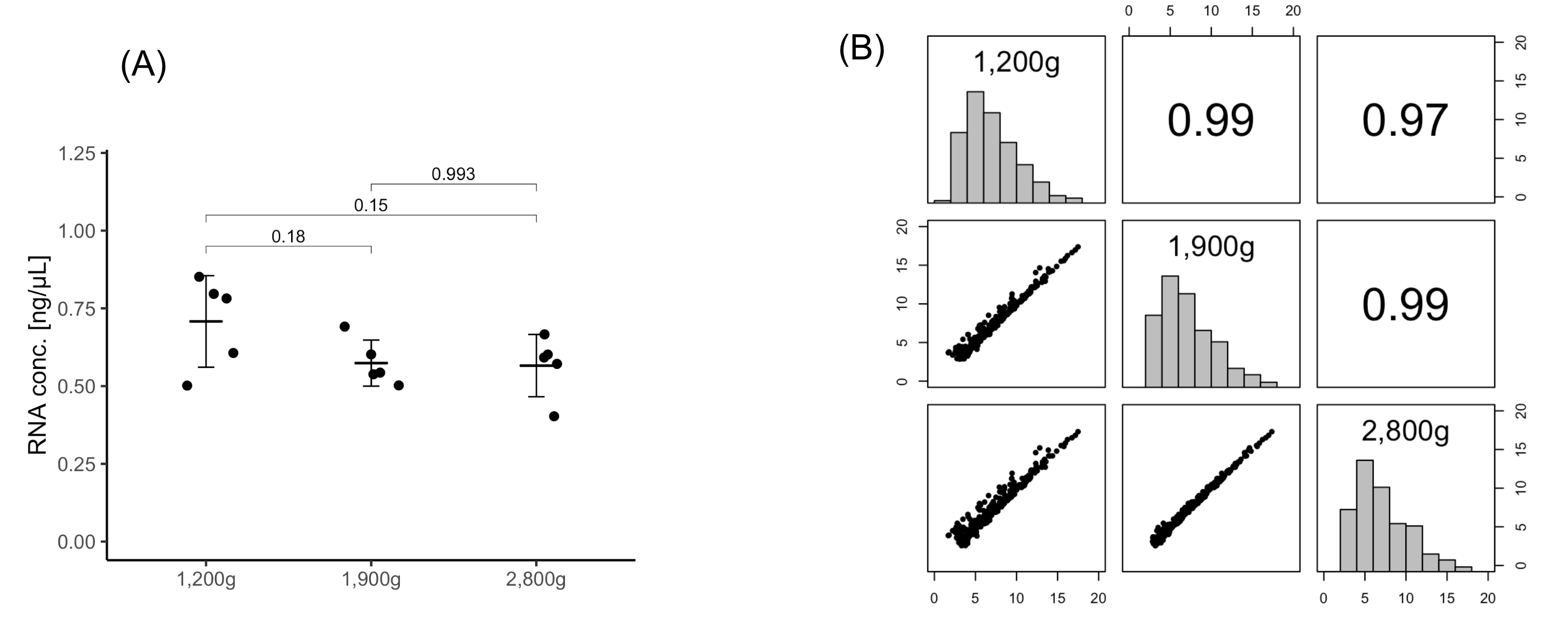

Supplement: S1 Fig — RNA concentration in samples obtained using different centrifugation conditions. Plasma was separated at 1,200 x g, 1,900 x g and 2,800 x g (A). Significant differences were identified using one-way ANOVA with a post hoc Tukey test and are indicated as * p<0.05, ** p<0.01 and *** p<0.001. Analysis of the correlation between miRNA expression levels in plasma. Plasma was separated at 1,200 x g, 1,900 x g and 2,800 x g. The expression levels are expressed as the mean of five different donor samples. MiRNAs with more than 8 TPM were selected and analyzed (B). (TIFF) [file pone.0278927.s001.tiff]

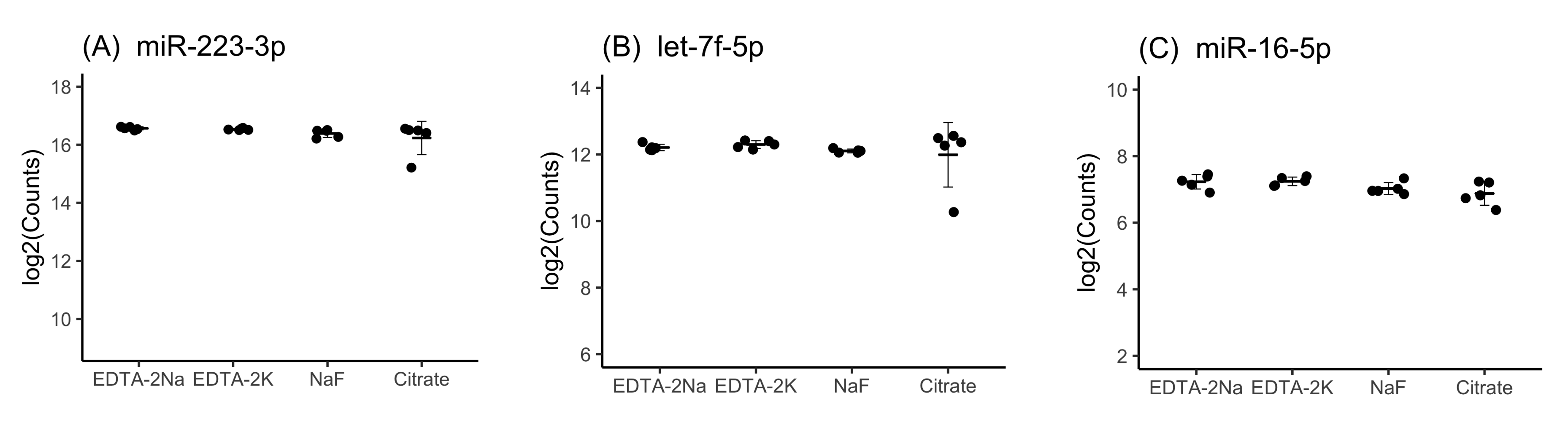

Supplement: S2 Fig — Hsa-miR-223-3p (A), hsa-let-7f-5p (B) and hsa-miR-16-5p (C) levels were evaluated in samples obtained in EDTA-2Na, EDTA-2K, NaF and citrate tubes. The crossbar indicates the mean, and the error bar indicates the standard deviation (SD). Significant differences were identified using one-way ANOVA with a post hoc Tukey test and are indicated as * p<0.05, ** p<0.01 and *** p<0.001. (TIFF) [file pone.0278927.s002.tiff]

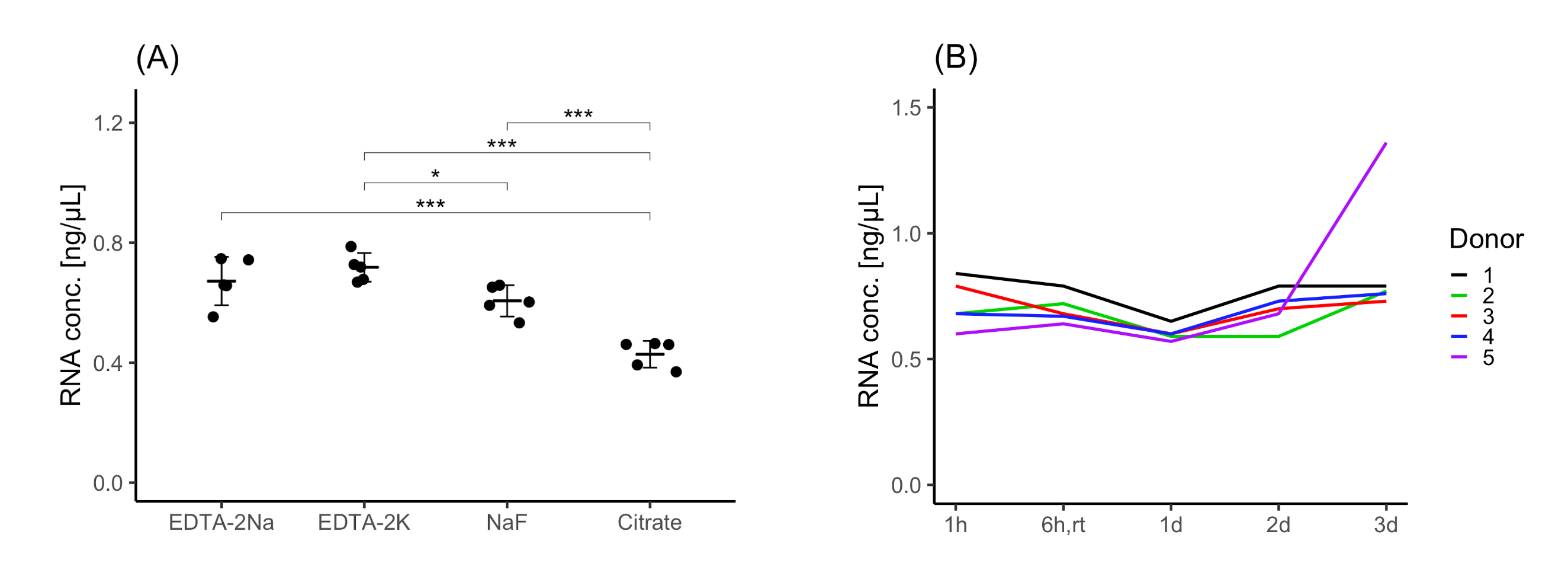

Supplement: S3 Fig — Concentration of RNAs obtained from samples isolated using different blood collection tube conditions (A). The crossbar indicates the mean, and the error bar indicates the standard deviation (SD). Significant differences were identified using one-way ANOVA with a post hoc Tukey test and are indicated as * p<0.05, ** p<0.01 and *** p<0.001. Time-dependent changes in RNA concentrations in whole blood stored at 4°C (B). Significant differences were identified using one-way ANOVA with a post hoc Dunnett’s test and are indicated as * p<0.05, ** p<0.01 and *** p<0.001. (TIFF) [file pone.0278927.s003.tiff]

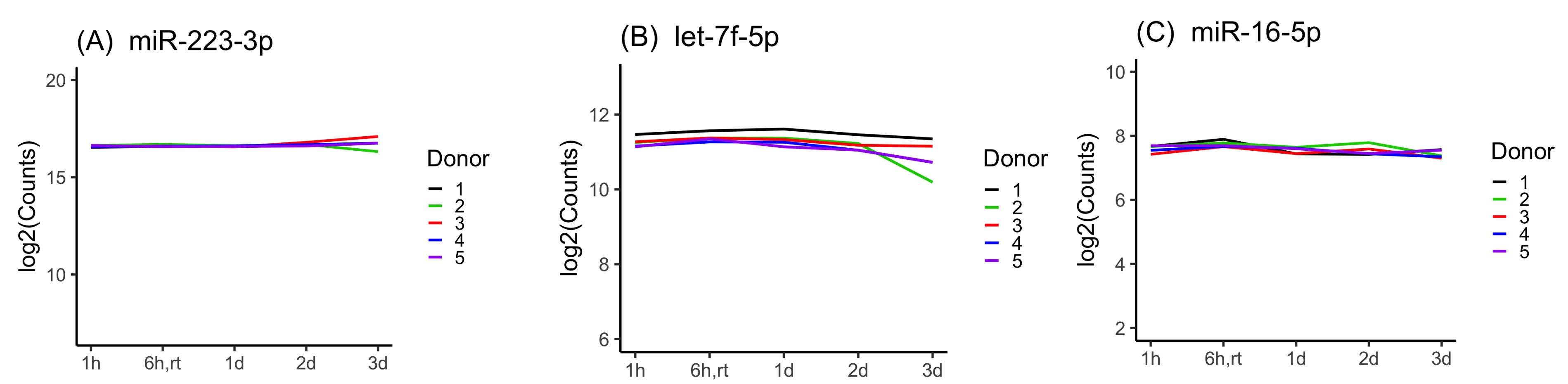

Supplement: S4 Fig — Hsa-miR-223-3p (A), hsa-let-7f-5p (B) and hsa-miR-16-5p (C) levels were evaluated at different times after blood collection: 1 hour, 6 hours (room temperature), 1 day, 2 days and 3 days. Significant differences were identified using one-way ANOVA with a post hoc Dunnett’s test and are indicated as * p<0.05, ** p<0.01 and *** p<0.001. (TIFF) [file pone.0278927.s004.tiff]

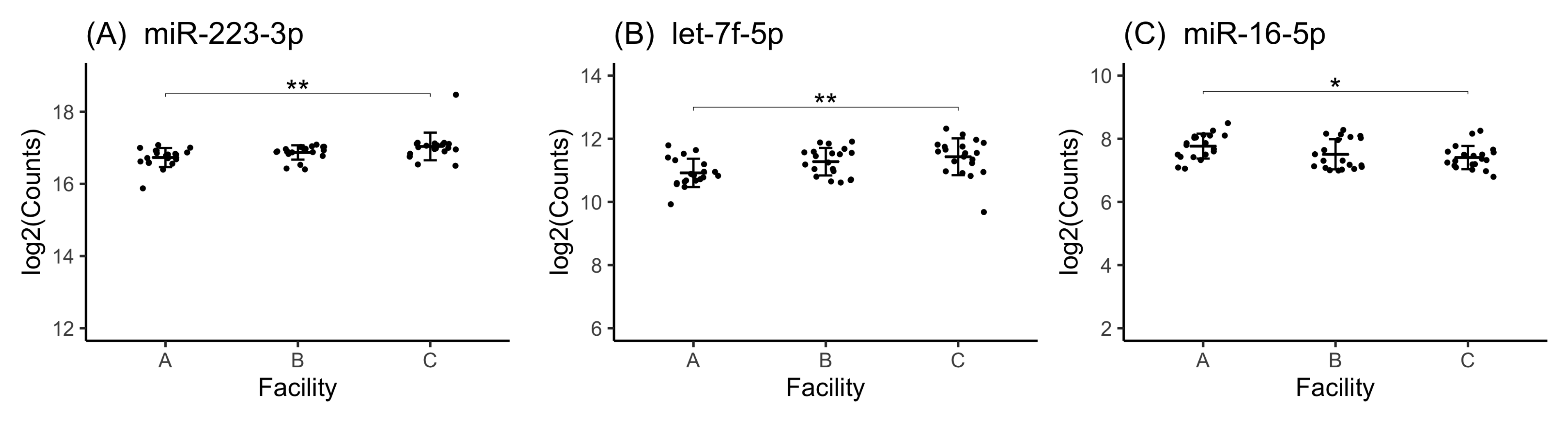

Supplement: S5 Fig — Hsa-miR-223-3p (A), hsa-let-7f-5p (B) and hsa-miR-16-5p (C) levels were compared among facilities. Significant differences were identified using one-way ANOVA with a post hoc Tukey test and are indicated as * p<0.05, ** p<0.01 and *** p<0.001. (TIFF) [file pone.0278927.s005.tiff]
